# Elicit

**DOI:** 10.29173/jchla29657

**Published:** 2023-04-01

**Authors:** Janice Y. Kung

**Affiliations:** Health Sciences Librarian University of Alberta Edmonton AB, Canada

**Product:** Elicit

**URL:** https://elicit.org/

## Product Description

Elicit is an online tool developed by Ought, a nonprofit machine learning (ML) research lab based in the United States. It is a free artificial intelligence (AI) research assistant that “uses language models to automate part of researchers’ workflows” [1]. Ideal for evidence synthesis and text extraction, Elicit pulls publications from Semantic Scholar and expedites the literature review process. Users enter a research question into the search box and the AI attempts to identify the top papers in the field. The AI can find relevant papers without perfect keyword matching, summarize takeaways from the paper, and extract key information into a research matrix. Taking inspiration from the systematic review process, the language model retrieves and condenses the information into component parts, thus allowing users to filter topics from a paper’s abstract including a shortened version of the abstract, intervention, outcomes, number of participants, population summary, and more. Elicit is ideal for questions that have empirical research (e.g., research in biomedicine) with interventions, randomized controlled trials, and questions generally structured as “What are the effects of x on y?” or “Does x affect y?” [2].

## Intended Users

Primarily used by researchers, Elicit is designed for students, independent researchers, and researchers from academic and independent organizations.

## Special Features

Elicit is dynamic. A summary of the top papers is presented along the left-hand navigation and is updated continuously as users remove irrelevant papers from the list. References may be starred and stored on a separate page for easy consultation at a later date.

**Fig. 1:**
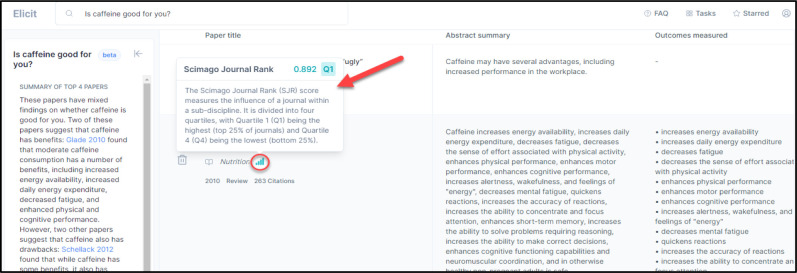
Sample SCImago Journal Rank display

The developers recently added the SCImago Journal Rank to publications as a way to quantify the prestige of a journal. By clicking on the graphical icon, the SCImago Journal Rank window appears with the score and quartile in which it belongs. In Figure 1, the paper has a ranking of 0.892 and falls in the first quartile, which means that the journal ranks in the top 25% of all journals from its sub-discipline. Other useful metadata are available including citation counts and a DOI link that redirects users to the original source.

**Fig. 2:**
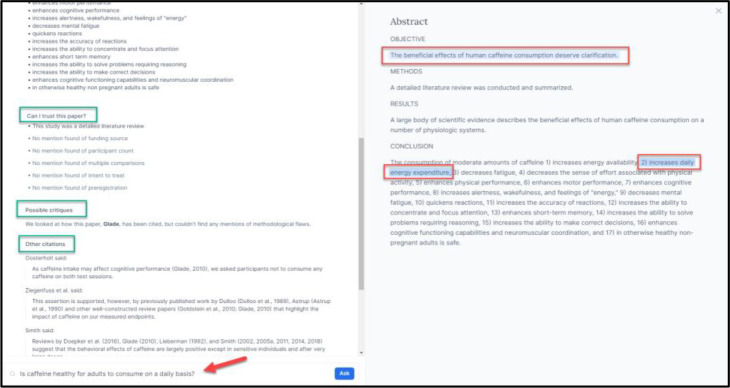
Sample question posed in an article

After clicking into a record, there are interesting insights that the AI pulls from the text (see Figure 2). They include:

- Abstract summary
- What did they test?
- Can I trust this paper?
- Possible critiques
- Other citations
- Search box for users to ask questions about the paper

The search box is another function that features the power of the AI tool by highlighting sections of the abstract that addresses the question posed (Figure 2).

**Fig. 3:**
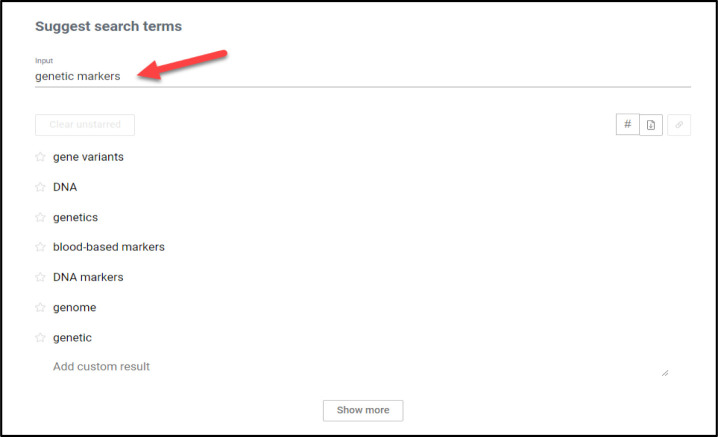
Suggest search terms example with “genetic markers”

In addition to exploring research questions (i.e., literature review), Elicit provides many other tools to help with the research process such as “Brainstorm research questions”, “Suggest search terms,” and “Abstract summarization”. These features are located under the Tasks section. Librarians and other information professionals may find “Suggest search terms” useful as it provides a list of synonyms for the term that was entered (Figure 3).

## Compatibility issues

Elicit works well across different web browsers (Chrome, Firefox, Microsoft Edge, and Safari).

## Usability

Elicit’s interface is simple and does not require a lot of training for new users to quickly grasp how the tool functions. Like Google, the main website presents a search box where users may “Ask a research question”. The Help page is also easy to find and navigate.

## Strengths

Elicit uses a process-based ML system and, when compared to other systems such as outcome-based systems, process-based ML systems provide “better differential capabilities” [3], whereby the system may perform tasks even though the outcomes are not accessible. It is a tool that streamlines the literature review process by retrieving eight papers most likely to answer the question, extracting, and summarizing important elements or variables based on user preference. References may be starred for easy retrieval at a later date. Since this is still a relatively new product, Elicit developers are regularly publishing enhancements and new features. They are receptive to feedback from end users.

## Weaknesses

Elicit is limited to publications in Semantic Scholar so there is a gap in the literature on what is being retrieved. For example, Semantic Scholar does not search licensed journals or behind paywalls [4] and a recent study in 2018 found that Google Scholar contained 389 million records while Semantic Scholar had 40 million records [5]. It does not have a search interface in the traditional sense; it is not designed to be searched by using keywords with search syntax (e.g., truncation) or controlled vocabulary. The machine encourages users to enter full research questions such as “How does iron supplementation affect anemia?” This could be a limitation since researchers need to ask the right questions for the right papers to be returned.

Users are unable to un-star items once they have been selected and there is no functionality to organize the starred items or to remove duplicates if the same reference has been starred from different search requests. After deleting papers from the results list, users are unable to retrieve them if they accidentally deleted relevant items. Some features on the website are not intuitive. For example, certain areas are clickable but users would not necessarily know unless they clicked around the page. If a user clicked on the title under the “Starred items,” they will be redirected to the Semantic Scholar page for the article, which is a useful feature that is not obvious.

## Comparison with similar products

There are several comparable AI tools that discover existing literature and summarize the content to save researchers time.

**ResearchRabbit** is a “citation-based literature mapping tool” [6] where users add one or many papers (seed papers) as the text corpus. The app will find similar papers and visualize connections between the seed papers and similar papers in network maps. ResearchRabbit is free and requires registered accounts from users so that researchers may set up alerts.

**Connected Papers** is similar to ResearchRabbit where it generates a network map of papers using co-citation and bibliographic coupling that matches the search criteria. Users may also toggle to view “Prior works” (papers that were most commonly cited by the papers in the graph) or “Derivative works” (papers that cited many papers in the graph). Publications in the graphs are also downloadable in a bib file. The free version of Connected Papers allows up to five graphs generated per month and does not require a registered account. It also includes different pricing models for academic and business users.

**Scholarcy** is an online article summarizer tool for articles, reports, and book chapters. It highlights key sections for users to easily save and export summaries to return to at a later date. Unlike Elicit, it can only summarize publications one at a time but extracts a lot more detailed information from the paper including key concepts, synopsis of the full-text, Scholarcy summary, comparative analysis, and more. Scholarcy comes with a free version with limited features.

## Cost

Elicit is currently free to use after the researcher creates an account.

## Conclusion

Elicit offers a unique tool that consolidates scholarly literature in a concise format to help users conduct literature reviews or find relevant articles to answer their research questions. While there are similar AI products previously mentioned, Elicit is novel in how it synthesizes literature and allows researchers to probe additional questions in an article.
